# Supplementary material for: Use of a glycomics array to establish the anti-carbohydrate antibody repertoire in type 1 diabetes
Source: Nat Commun. 2022 Nov 1;13:6527. doi: 10.1038/s41467-022-34341-2 (PMC9622713; doi:10.1038/s41467-022-34341-2)
Supplement: Supplementary file 1 — Supplementary Information [file 41467_2022_34341_MOESM1_ESM.pdf]

Supplementary Information for "Use of a Glycomics Array to establish the Anti-carbohydrate antibody repertoire in type 1 diabetes"

Paul MH Tran[1], Fran Dong[2], Eileen Kim[1], Katherine P. Richardson[1], Lynn KH Tran[1], Kathleen Waugh[2], Diane Hopkins[1], Richard D. Cummings[3], Peng George Wang[4], Marian Rewers[2], Jin-Xiong She[1], and Sharad Purohit[1,5,6]

Table of Contents

Title	Page(s)
Supplementary Table 1: PAGODA FUT2 genotype clinical characteristics	3
Supplementary Figure 1: Dynamic range of the glycan array	4
Supplementary Figure 2: Comparison of single-plex vs multiplex glycan array	5
Supplementary Figure 3: Assessment of signal-to-noise ratio (SNR) for analysis of anti-carbohydrate antibodies	6
Supplementary Figure 4: Comparing human serum albumin (HSA) vs bovine serum albumin (BSA) as blocker for glycan array	7
Supplementary figure 5: Intra- and Inter-assay reproducibility.	8
Supplementary Figure 6: Dynamic range and specificity determined by anti-Kanamycin antibody	9
Supplementary Figure 7: Evaluating stability of anti-carbohydrate IgG in serum samples	10
Supplementary Figure 8: Correlation heatmap of ACA clusters	11
Supplementary Figure 9: Radar Chart of Mono-, di-, and trisaccharides	12
Supplementary Figure 10: Density distribution of MFI of ACAs against gentamicin in DAISY cohort	13
Information on R-session used for running the analysis for generation of figures in the paper	14-16
R Packages citations	17-18

Supplementary Table 1. PAGODA FUT2 genotype clinical characteristics

Characteristic	**Homozygous G/G**, N = 41[1]	**Homozygous A/A**, N = 39[1]	**Heterozygous G/A**, N = 84[1]	**p-value**[2]
Group				>0.9
Control	14 (34%)	14 (36%)	32 (38%)	
Progressor	27 (66%)	25 (64%)	52 (62%)	
Sex				0.6
Female	22 (54%)	25 (64%)	52 (62%)	
Male	19 (46%)	14 (36%)	32 (38%)	
FDR	13 (32%)	12 (31%)	29 (35%)	>0.9
Draw_Age	39 (23, 45)	41 (21, 48)	32 (16, 44)	0.13
HLA_risk				0.9
H	10 (26%)	8 (23%)	20 (27%)	
L	28 (74%)	27 (77%)	53 (73%)	
Unknown	3	4	11	

[1] n (%); Median (IQR)
[2] Pearson's Chi-squared test; Kruskal-Wallis rank sum test

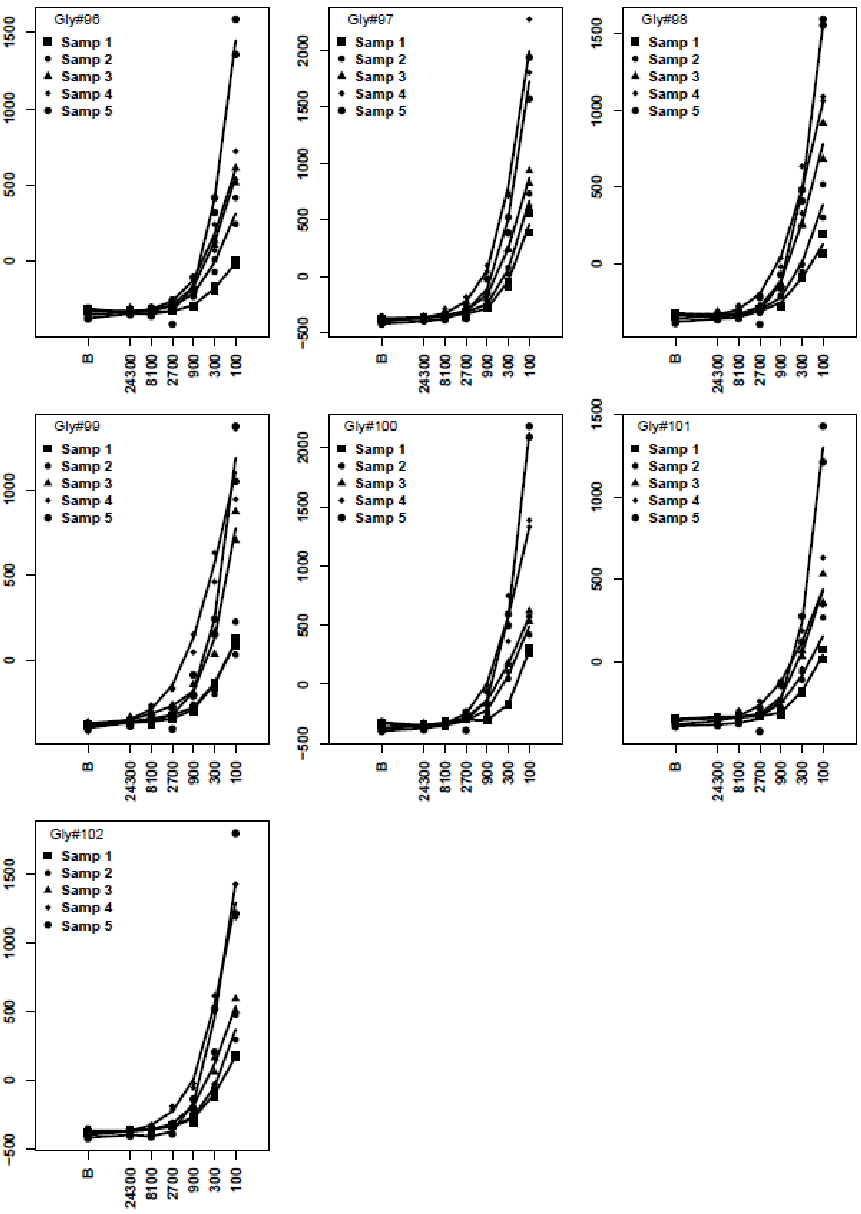

Supplementary Figure 1: Dynamic range of the glycan array determined by assaying 3-fold serum dilution series (100-,300-,900-,2700-,8100-,24300-fold). Serum samples (n=5) were selected based on their highest median fluorescent intensities observed for gentamicin. Gly#96: Tobramicin, Gly#97: Amikacin, Gly#98: Gentamicin Sulfate, Gly#99: Kanamycin sulfate, Gly#100: Geneticin Disulfate salt (G418), Gly#101: Neomycin trisulfate, Gly#102: Sisomicin. Background corrected median fluorescent intensity (MFI, y-axis) is plotted against dilution factor for serum (x-axis). The dynamic range of the assay was evaluated by modeling MFI values from 100-, 300-, 900- and 2700-fold diluted serum. The dilution series were converted into percentage of serum to perform the linear regression. R-squared (R^2) was taken as measure of linearity from the linear regression. For all the seven-aminoglycosides, the R^2 values obtained for all the samples and aminoglycosides were averaged and the range was determined. In this regression analysis, the average R^2 was 0.96 (0.83-0.99).

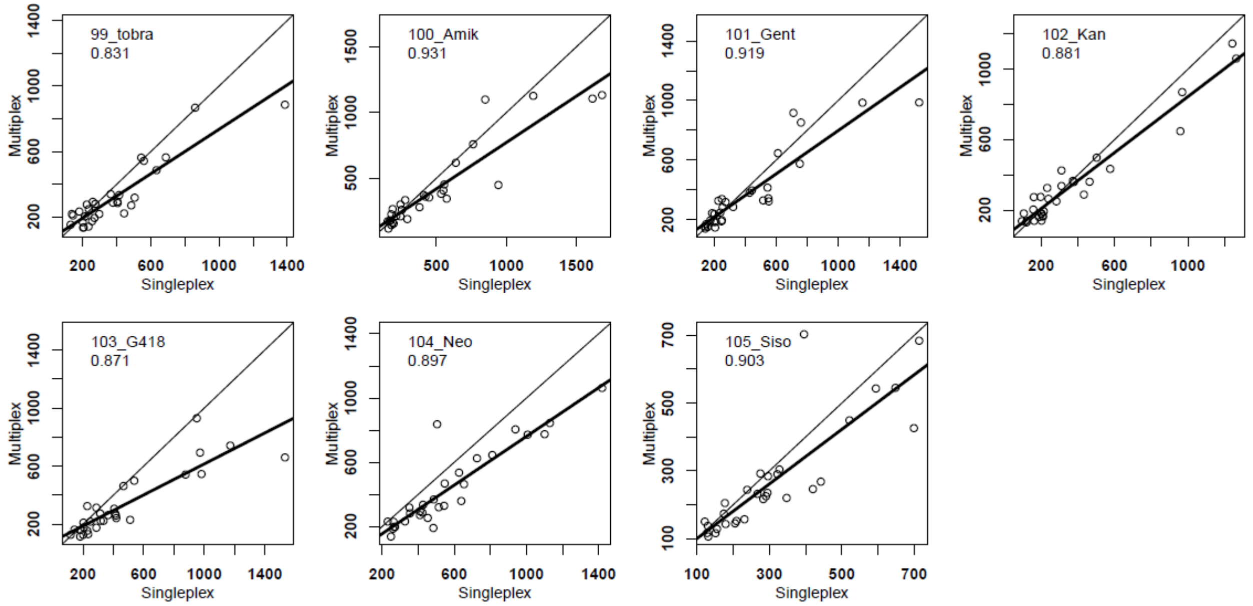

Supplementary Figure 2: Pairs plots showing the comparison of single-plex vs multiplex for measurement of anti-aminoglycoside antibodies in the serum of human subjects (n=5). Three-fold dilution series (100-, 300-, and 900-fold) were analyzed for comparison. Correlation coefficient (r) was used as a measure to evaluate the differences in binding of anti-carbohydrate IgG in a single-plex and multiplex assay. The average correlation coefficient was 0.89, the r values ranged from 0.831-0.931.

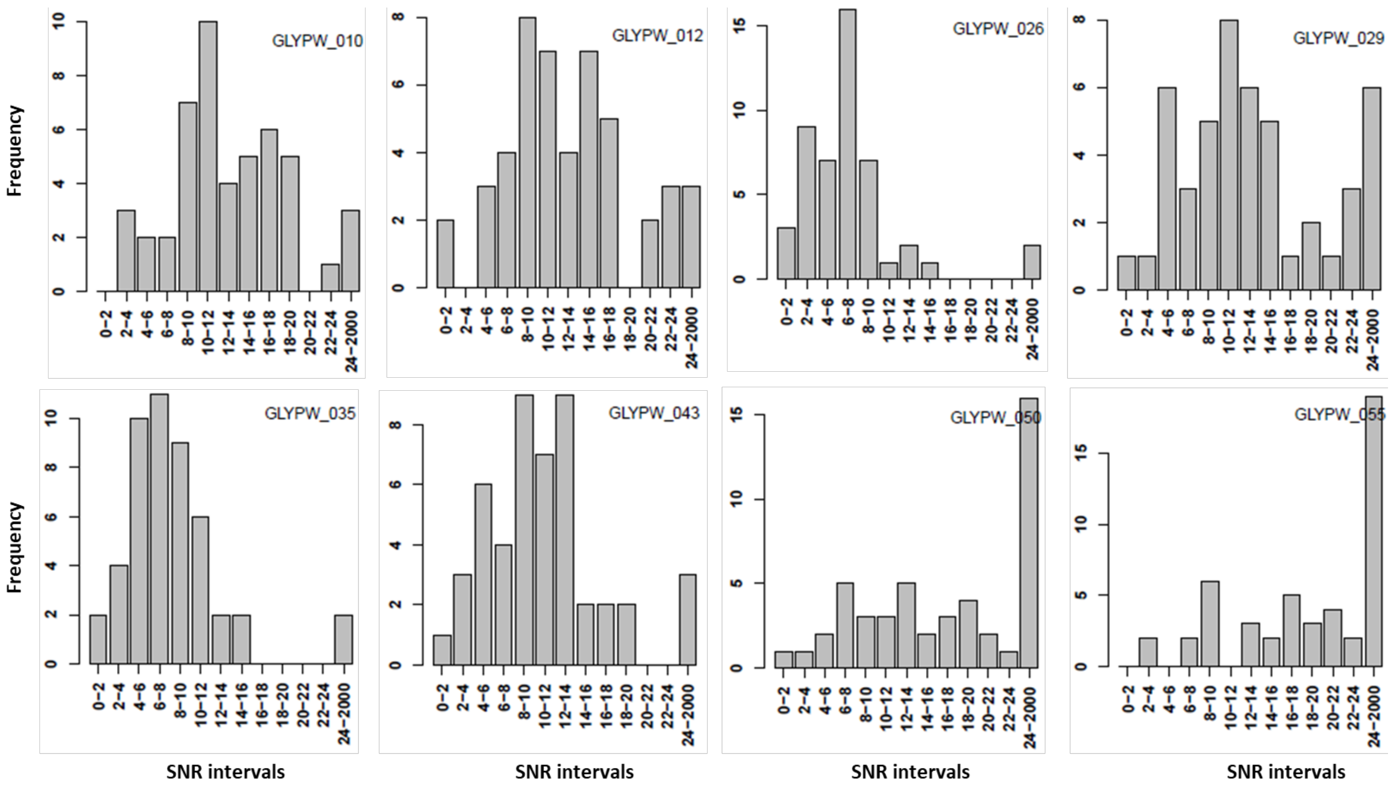

Supplementary Figure 3: Assessment of signal-to-noise ratio (SNR) for analysis of anti-carbohydrate antibodies present in serum. Signal-to-noise ratio analysis was perform using serum samples (n=48) on a 45-plex array consisting of mono-, di-, tri- and other complex glycans. Serum samples were diluted 500-fold with phosphate buffer pH7.4, containing 1% human serum albumin and 250mM sodium chloride. Signal to noise ratio was determined by dividing the median fluorescence intensity (MFI) of the glycan bead by the average MFI of the three no glycan conjugation (NGC) control beads. Frequency of the SNR values (y-axis) for each glycan is plotted against the interval of SNR (x-axis). Representative plots are presented.

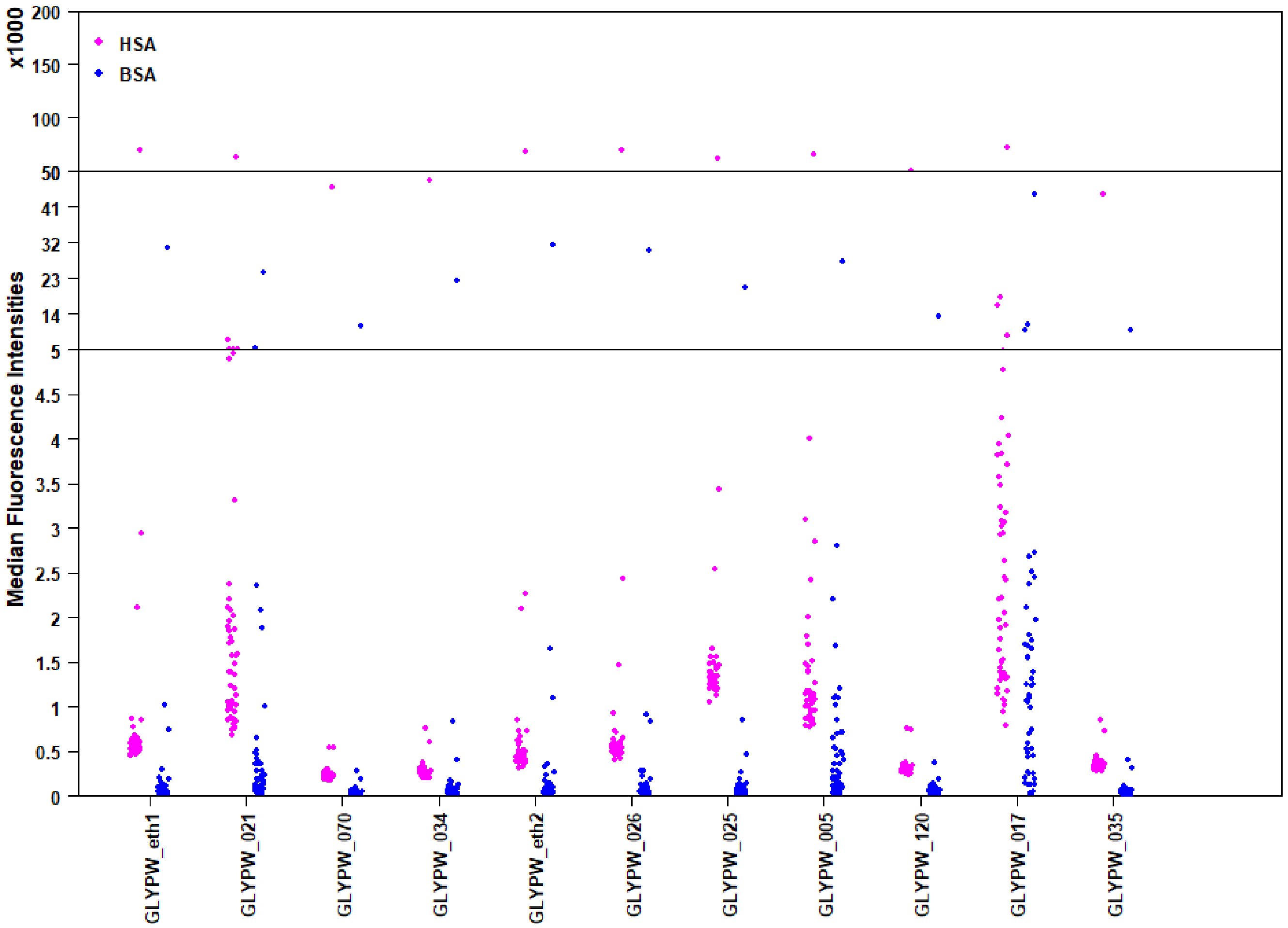

Supplementary Figure 4: Comparison human serum albumin (HSA) and bovine serum albumin (BSA) as blocker for beads and for dilution of serum for analysis of anti-carbohydrate immunoglobulin M (IgM). Selected glycans representing mono-, di, tri-saccharides and other branched glycans were evaluated for the binding of anti-carbohydrate IgM to test the blockers for conjugated beads. Conjugation of the glycans was performed as reported in the methods sections. The conjugated beads were blocked with 1% HSA (wt/v) or with 1% BSA (wt/v) in phosphate buffer containing 250mM sodium chloride (pH7.4). Serum samples (n=32) were diluted 500-fold and then analyzed on the blocked beads. Based on the signals for selected glycans, HSA was chosen over BSA for blocking beads and diluent for measuring IgG/M type of anti-carbohydrate antibodies in human serum samples.

a)

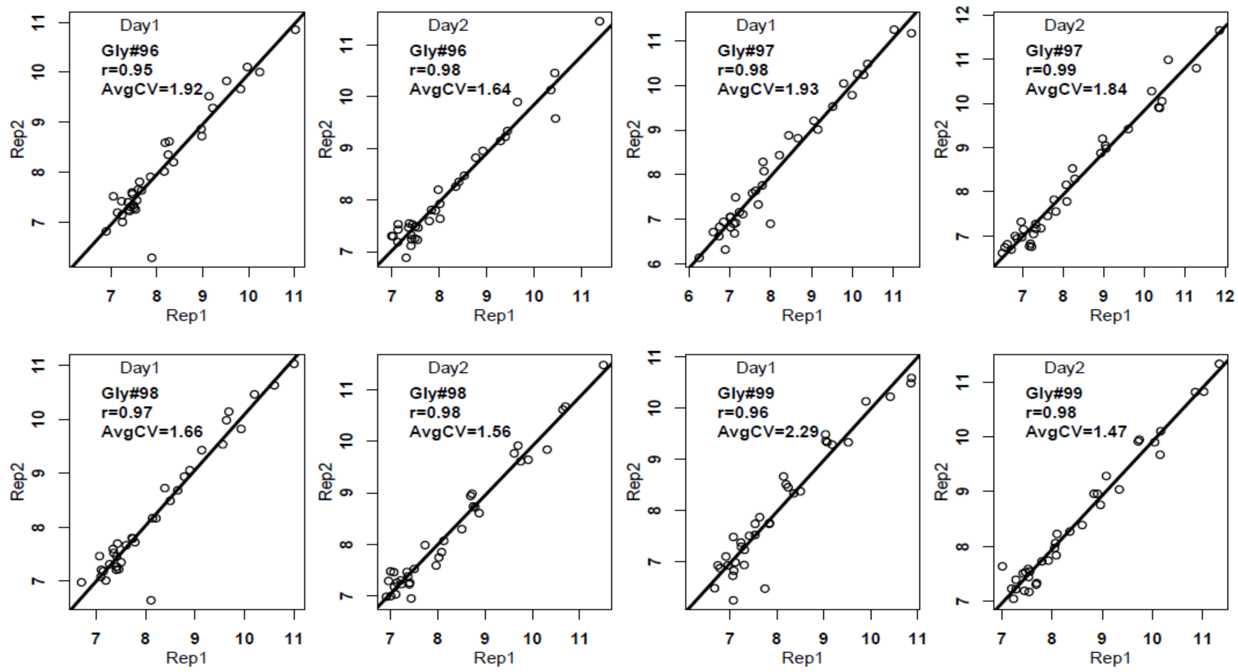

b)

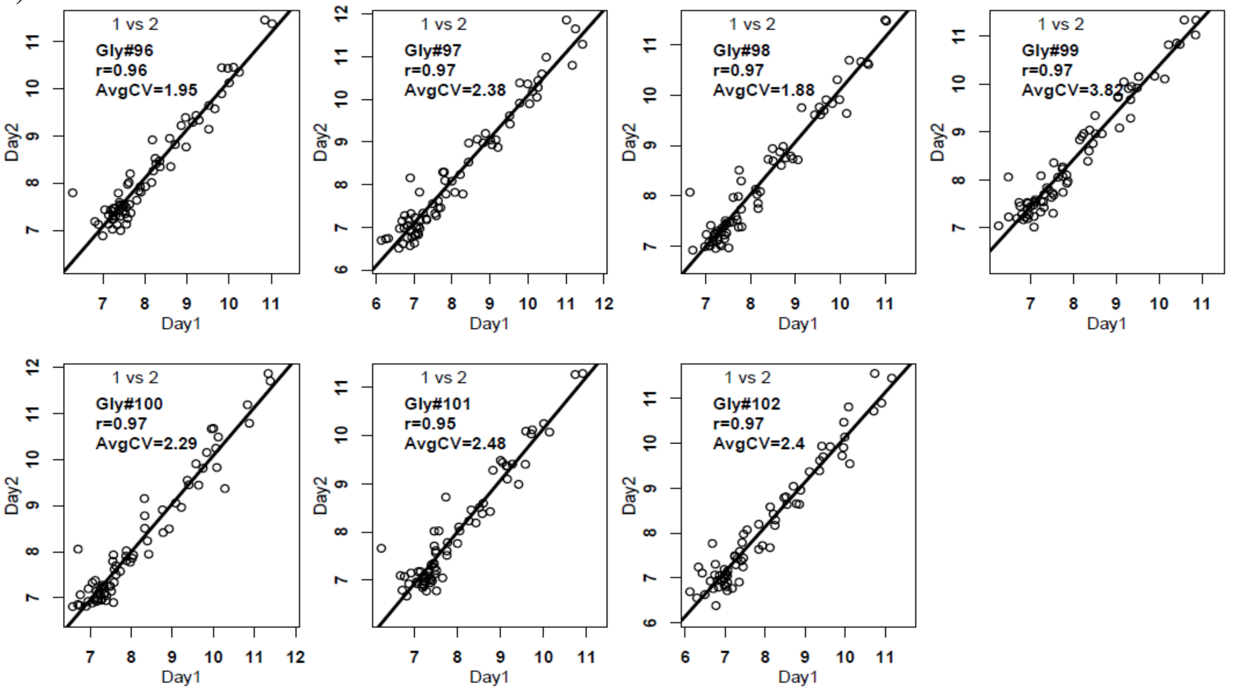

Supplementary Figure 5: Plots showing intra- (a) and inter-day (b) reproducibility of the anti-aminoglycoside antibodies present in human serum (n=5). A three-fold dilution curve was run on two separate days for aminoglycoside beads to evaluate the reproducibility. Prior to any analysis median fluorescence intensities were log2 transformed. Intra-day reproducibility was assessed by determining coefficient of variation (CV) and correlation coefficient between two replicate wells on each day. Inter-day reproducibility was assessed by determining the CV and r values between two different days.

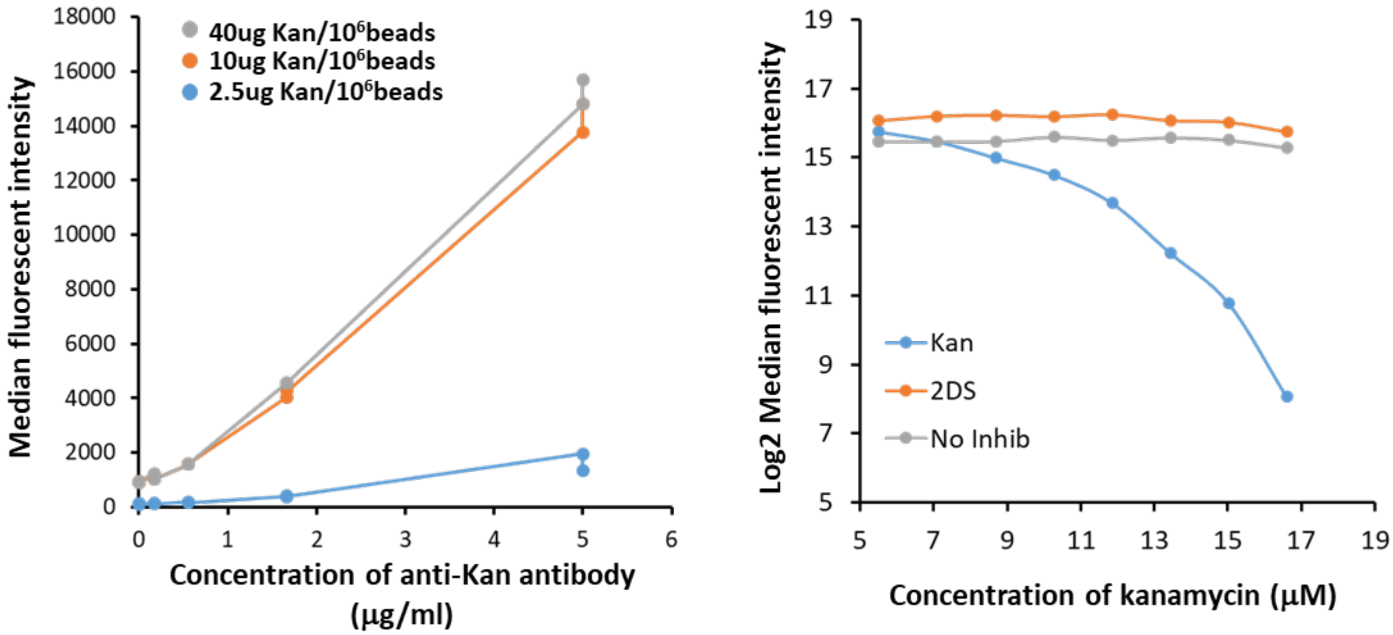

Supplementary Figure 6: Dynamic range and specificity of amino-glycoside beads in glycan array determined by assaying 3 fold dilution of anti-kanamycin antibody (5ug/ml).**A:** Background corrected median fluorescence intensity (MFI, y-axis) is plotted against 3-fold dilution of anti-kanamycin antibody (x-axis). The antibody binding was tested on beads conjugated with 3 different concentration of kanamycin viz., 2.5µg/10^6 beads (blue line), 10 µg/10^6 beads (orange line) and 40 µg/10^6 beads (gray line). **B:** Specificity of beads was tested by inhibiting the binding of anti-kanamycin antibody to kanamycin conjugated beads in presence of 3-fold dilution of 100µM kanamycin. Log2 transformed background corrected MFI (y-axis) is plotted against log2 concentration of kanamycin as inhibitor. Blue line represents binding of anti-kanamycin (2ug/ml) antibody in presence of increasing concentration of kanamycin. Orange line represents binding of anti-kanamycin (2ug/ml) antibody to the kanamycin beads in presence of 2-Deoxystreptamine (2DS). Gray line represents kanamycin binding of anti-kanamycin (2ug/ml) antibody to beads.

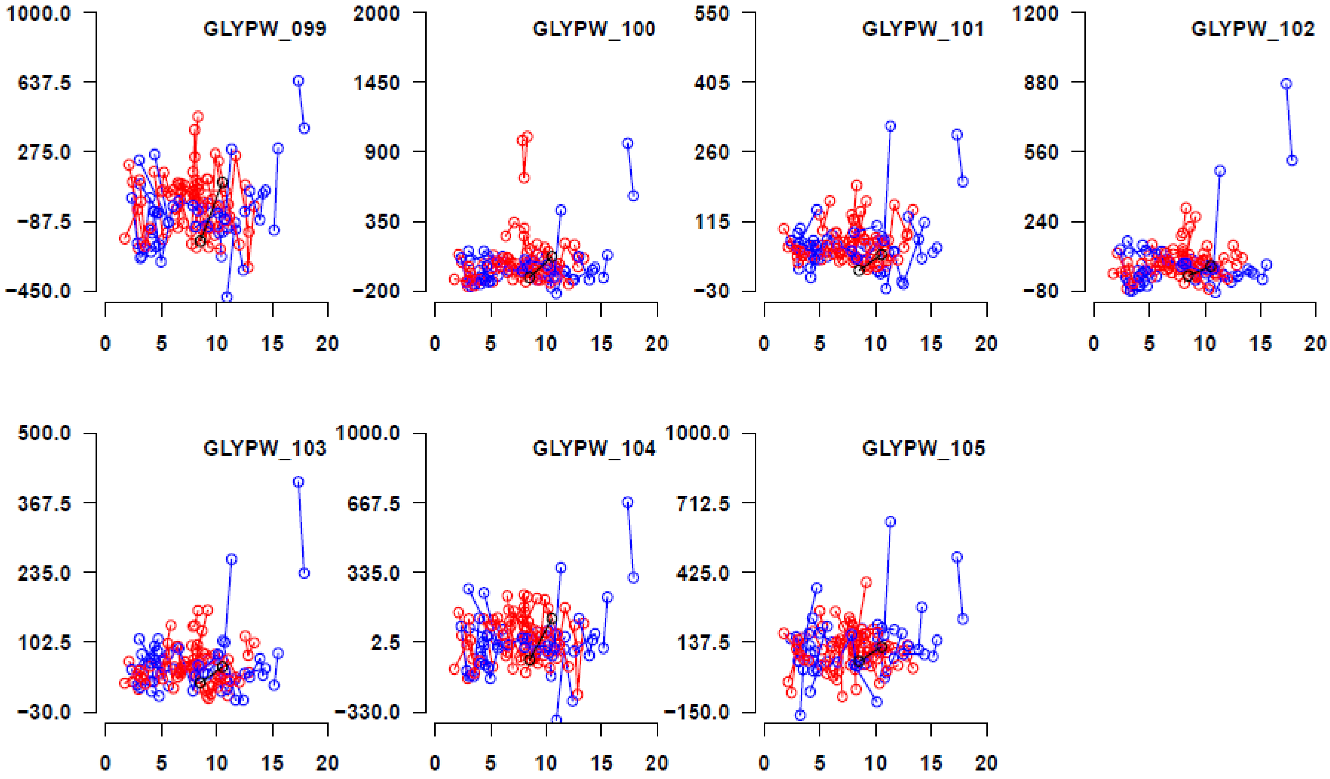

Supplementary Figure 7: Representative line plots for aminoglycosides evaluating stability of anti-carbohydrate IgG in serum samples from DAISY subjects (n=112). We selected data for seven aminoglycosides data to represent here. Background corrected MFI (y-axis) were plotted against age of sample collection (x-axis) to show the stability of the glycan array. Serum samples (n=57) from DAISY subjects were collected at multiple time points (2-4) and analyzed to evaluate the stability of the measurement of anti-carbohydrate IgG in serum. Serum from type 1 diabetes patients (red circles and line) and controls (blue circles and line) were analyzed on the glycan array.

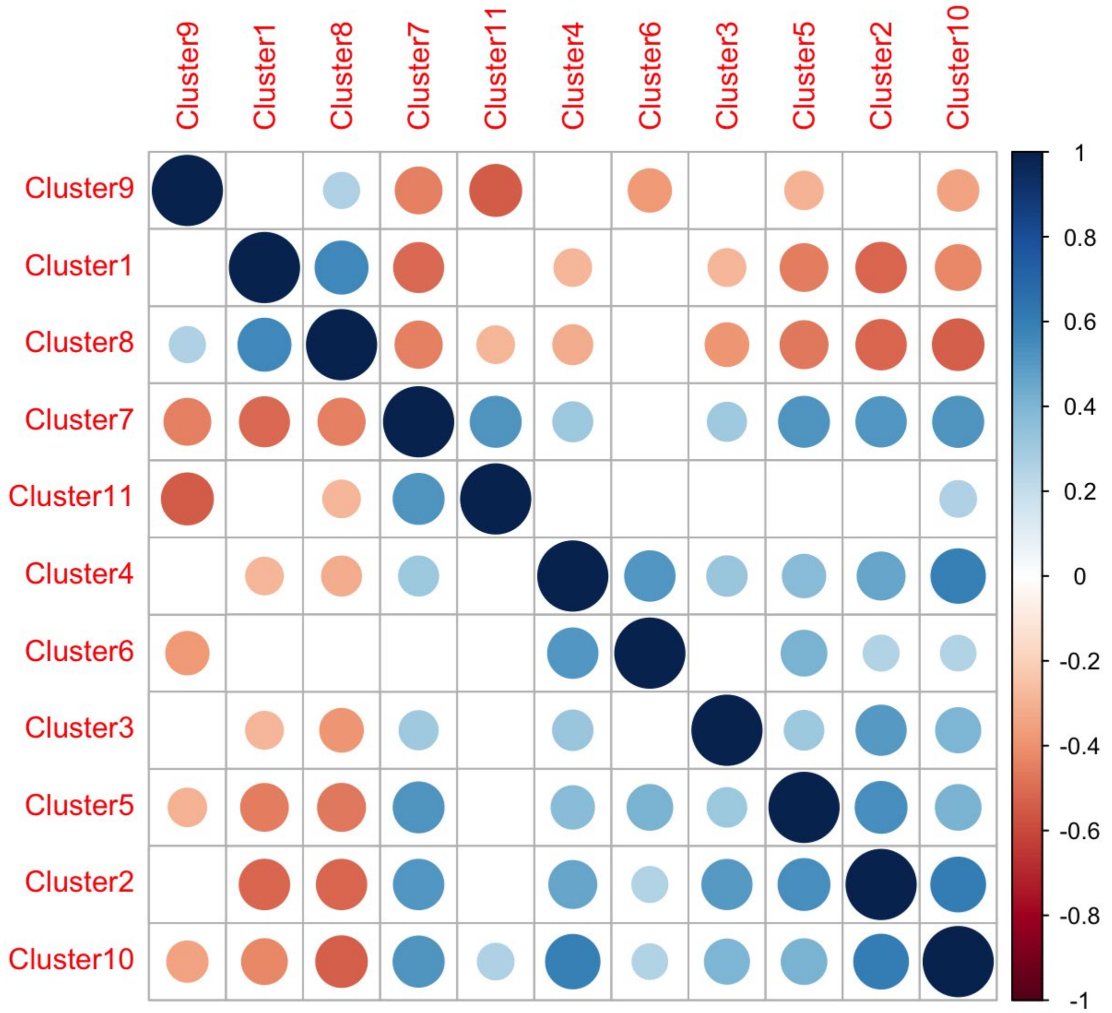

Supplementary Figure 8. Correlation heatmap of ACA clusters

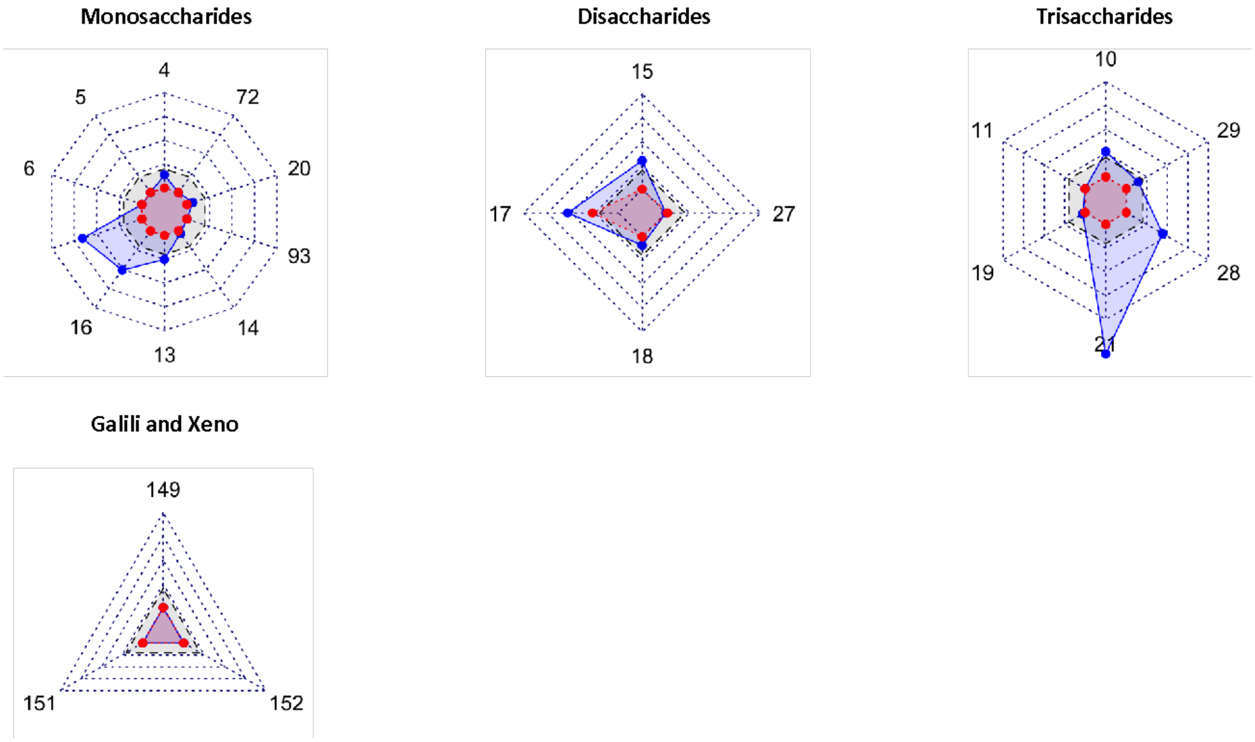

Supplementary Figure 9. Radar Chart of Mono-, di-, and trisaccharides associated with islet autoimmunity and T1D progression

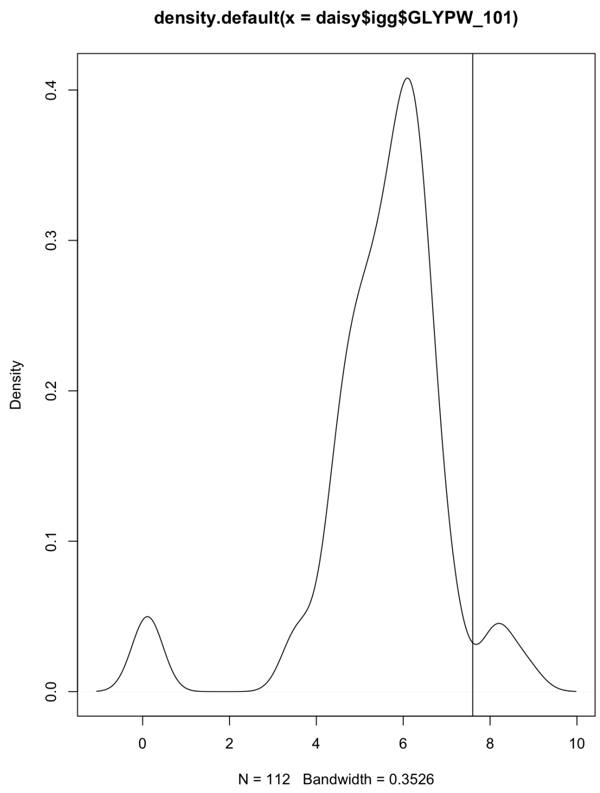

Supplementary Figure 10: Density distribution of MFI of ACAs against gentamicin in DAISY cohort

Information on R-session used for running the analysis for generation of figures in the paper

```
> sessionInfo()
R version 4.1.2 (2021-11-01)
Platform: x86_64-apple-darwin17.0 (64-bit)
Running under: macOS Big Sur 11.6

Matrix products: default
LAPACK: /Library/Frameworks/R.framework/Versions/4.1/Resources/lib/libRlapack.dylib

locale:
[1] en_US.UTF-8/en_US.UTF-8/en_US.UTF-8/C/en_US.UTF-8/en_US.UTF-8

attached base packages:
[1] grid      stats     graphics  grDevices
[5] utils     datasets  methods   base

other attached packages:
 [1] corrplot_0.92         glmnet_4.1-3
 [3] Matrix_1.3-4          fmsb_0.7.2
 [5] pROC_1.18.0           BiocManager_1.30.16
 [7] Ryacas_1.1.3.1        circlize_0.4.14
 [9] dendextend_1.15.2     gtsummary_1.5.2
[11] gmodels_2.18.1        dplyr_1.0.8
[13] fastDummies_1.6.3     Hmisc_4.6-0
[15] Formula_1.2-4         survival_3.2-13
[17] lattice_0.20-45       alluvial_0.1-2
[19] igraph_1.2.11         umap_0.2.7.0
[21] xlsx_0.6.5            ComplexHeatmap_2.11.1
[23] PCAtools_2.6.0        ggrepel_0.9.1
[25] ggplot2_3.3.5         pacman_0.5.1

loaded via a namespace (and not attached):
  [1] colorspace_2.0-3
  [2] rjson_0.2.21
  [3] ellipsis_0.3.2
  [4] rprojroot_2.0.2
  [5] htmlTable_2.4.0
  [6] GlobalOptions_0.1.2
  [7] base64enc_0.1-3
  [8] clue_0.3-60
  [9] rstudioapi_0.13
 [10] RSpectra_0.16-0
 [11] fansi_1.0.2
 [12] codetools_0.2-18
 [13] splines_4.1.2
 [14] sparseMatrixStats_1.6.0
 [15] doParallel_1.0.17
 [16] knitr_1.37
```

[17] pkgload_1.2.4
[18] jsonlite_1.8.0
[19] gt_0.4.0
[20] broom_0.7.12
[21] rJava_1.0-6
[22] cluster_2.1.2
[23] png_0.1-7
[24] compiler_4.1.2
[25] dqrng_0.3.0
[26] backports_1.4.1
[27] assertthat_0.2.1
[28] fastmap_1.1.0
[29] cli_3.2.0
[30] BiocSingular_1.10.0
[31] htmltools_0.5.2
[32] tools_4.1.2
[33] rsvd_1.0.5
[34] gtable_0.3.0
[35] glue_1.6.1
[36] reshape2_1.4.4
[37] Rcpp_1.0.8
[38] vctrs_0.3.8
[39] gdata_2.18.0
[40] broom.helpers_1.6.0
[41] iterators_1.0.14
[42] DelayedMatrixStats_1.16.0
[43] lmtest_0.9-39
[44] xfun_0.29
[45] stringr_1.4.0
[46] brio_1.1.3
[47] xlsxjars_0.6.1
[48] testthat_3.1.2
[49] beachmat_2.10.0
[50] lifecycle_1.0.1
[51] irlba_2.3.5
[52] gtools_3.9.2
[53] MASS_7.3-54
[54] zoo_1.8-9
[55] scales_1.1.1
[56] MatrixGenerics_1.6.0
[57] parallel_4.1.2
[58] RColorBrewer_1.1-2
[59] reticulate_1.24
[60] gridExtra_2.3
[61] sass_0.4.0
[62] rpart_4.1-15
[63] latticeExtra_0.6-29
[64] stringi_1.7.6

[65] desc_1.4.0
[66] S4Vectors_0.32.3
[67] foreach_1.5.2
[68] ScaledMatrix_1.2.0
[69] checkmate_2.0.0
[70] BiocGenerics_0.40.0
[71] BiocParallel_1.28.3
[72] shape_1.4.6
[73] commonmark_1.7
[74] rlang_1.0.1
[75] pkgconfig_2.0.3
[76] matrixStats_0.61.0
[77] purrr_0.3.4
[78] htmlwidgets_1.5.4
[79] cowplot_1.1.1
[80] tidyselect_1.1.2
[81] plyr_1.8.6
[82] magrittr_2.0.2
[83] R6_2.5.1
[84] IRanges_2.28.0
[85] generics_0.1.2
[86] DelayedArray_0.20.0
[87] DBI_1.1.2
[88] pillar_1.7.0
[89] foreign_0.8-81
[90] withr_2.4.3
[91] nnet_7.3-16
[92] tibble_3.1.6
[93] crayon_1.5.0
[94] utf8_1.2.2
[95] viridis_0.6.2
[96] jpeg_0.1-9
[97] GetoptLong_1.0.5
[98] data.table_1.14.2
[99] forcats_0.5.1
[100] digest_0.6.29
[101] tidyr_1.2.0
[102] openssl_1.4.6
[103] stats4_4.1.2
[104] munsell_0.5.0
[105] viridisLite_0.4.0
[106] askpass_1.1

Supplementary References

1. Rinker TW, Kurkiewicz D (2018). _pacman: Package Management for R_. version 0.5.0,<URL: http://github.com/trinker/pacman>.

2. Blighe K, Lun A (2021). _PCAtools: PCAtools: Everything Principal Components Analysis_. R package version 2.6.0, <URL: https://github.com/kevinblighe/PCAtools>.

3. Gu Z, Eils R, Schlesner M (2016). "Complex heatmaps reveal patterns and correlations in multidimensional genomic data." _Bioinformatics_.

4. Dragulescu A, Arendt C (2020). _xlsx: Read, Write, Format Excel 2007 and Excel 97/2000/XP/2003 Files_. R package version 0.6.5, <URL: https://CRAN.R-project.org/package=xlsx>.

5. Konopka T (2020). _umap: Uniform Manifold Approximation and Projection_. R package version 0.2.7.0, <URL: https://CRAN.R-project.org/package=umap>.

6. Csardi G, Nepusz T (2006). "The igraph software package for complex network research." _InterJournal_, *Complex Systems*, 1695. <URL: https://igraph.org>.

7. Wickham H (2016). _ggplot2: Elegant Graphics for Data Analysis_. Springer-Verlag New York. ISBN 978-3-319-24277-4, <URL: https://ggplot2.tidyverse.org>.

8. Bojanowski M, Edwards R (2016). _alluvial: R Package for Creating Alluvial Diagrams_. R package version: 0.1-2, <URL: https://github.com/mbojan/alluvial>.

9. Harrell Jr F (2021). _Hmisc: Harrell Miscellaneous_. R package version 4.6-0, <URL: https://CRAN.R-project.org/package=Hmisc>.

10. Kaplan J (2020). _fastDummies: Fast Creation of Dummy (Binary) Columns and Rows from Categorical Variables_. R package version 1.6.3, <URL: https://CRAN.R-project.org/package=fastDummies>.

11. Wickham H, François R, Henry L, Müller K (2022). _dplyr: A Grammar of Data Manipulation_. R package version 1.0.8, <URL: https://CRAN.R-project.org/package=dplyr>.

12. Warnes GR, Bolker B, Lumley T, SAIC-Frederick RCJCfRCJaC, Program IFbtIR, NIH ot, Institute NC, NO1-CO-12400.fCRuNC (2018). _gmodels: Various R Programming Tools for Model Fitting_. R package version 2.18.1, <URL: https://CRAN.R-project.org/package=gmodels>.

13. Sjoberg D, Whiting K, Curry M, Lavery J, Larmarange J (2021). "Reproducible Summary Tables with the gtsummary Package." _The R Journal_, *13*, 570-580. doi: 10.32614/RJ-2021-053 (URL: https://doi.org/10.32614/RJ-2021-053), <URL:https://doi.org/10.32614/RJ-2021-053>.

14. Galili T (2015). “dendextend: an R package for visualizing, adjusting, and comparing trees of hierarchical clustering.” _Bioinformatics_. doi: 10.1093/bioinformatics/btv428 (URL: https://doi.org/10.1093/bioinformatics/btv428), https://academic.oup.com/bioinformatics/article-pdf/31/22/3718/17122682/btv428.pdf,
15. <URL: https://academic.oup.com/bioinformatics/article/31/22/3718/240978/dendextend-an-R-package-for-visualizing-adjusting>.

16. Gu Z, Gu L, Eils R, Schlesner M, Brors B (2014). “circlize implements and enhances circular visualization in R.” _Bioinformatics_, *30*, 2811-2812.

17. Andersen M, Højsgaard S (2019). “Ryacas: A computer algebra system in R.” _Journal of Open Source Software_, *4*(42). <URL: https://doi.org/10.21105/joss.01763>.

18. Morgan M (2021). _BiocManager: Access the Bioconductor Project Package Repository_. R package version 1.30.16, <URL: https://CRAN.R-project.org/package=BiocManager>.

19. Robin X, Turck N, Hainard A, Tiberti N, Lisacek F, Sanchez J, Müller M (2011). “pROC: an open-source package for R and S+ to analyze and compare ROC curves.” _BMC Bioinformatics_, *12*, 77.

20. Nakazawa M (2021). _fmsb: Functions for Medical Statistics Book with some Demographic Data_. R package version 0.7.2, <URL: https://CRAN.R-project.org/package=fmsb>.

21. Friedman J, Hastie T, Tibshirani R (2010). “Regularization Paths for Generalized Linear Models via Coordinate Descent.” _Journal of Statistical Software_, *33*(1), 1-22. <URL: https://www.jstatsoft.org/v33/i01/>.

22. Simon N, Friedman J, Hastie T, Tibshirani R (2011). “Regularization Paths for Cox's Proportional Hazards Model via Coordinate Descent.” _Journal of Statistical Software_, *39*(5), 1-13. <URL:https://www.jstatsoft.org/v39/i05/>.

23. Wei T, Simko V (2021). _R package 'corrplot': Visualization of a Correlation Matrix_. (Version 0.92), <URL: https://github.com/taiyun/corrplot>.
