# Supplementary material for: Use of a glycomics array to establish the anti-carbohydrate antibody repertoire in type 1 diabetes
Source: Nat Commun. 2022 Nov 1;13:6527. doi: 10.1038/s41467-022-34341-2 (PMC9622713; doi:10.1038/s41467-022-34341-2)
Supplement: Supplementary file 3 — Description of Additional Supplementary Files [file 41467_2022_34341_MOESM3_ESM.pdf]

Description of Additional Supplementary Files

Supplementary Data 1: This file contains glycan structures used to generate the glycan array
