# Supplementary material for: Use of a glycomics array to establish the anti-carbohydrate antibody repertoire in type 1 diabetes
Source: Nat Commun. 2022 Nov 1;13:6527. doi: 10.1038/s41467-022-34341-2 (PMC9622713; doi:10.1038/s41467-022-34341-2)
Supplement: Supplementary file 5 — Reporting Summary [file 41467_2022_34341_MOESM5_ESM.pdf]

nature portfolio

Corresponding author(s): Sharad Purohit

Last updated by author(s): Oct 4, 2022

Reporting Summary

Nature Portfolio wishes to improve the reproducibility of the work that we publish. This form provides structure for consistency and transparency in reporting. For further information on Nature Portfolio policies, see our Editorial Policies and the Editorial Policy Checklist.

Statistics

For all statistical analyses, confirm that the following items are present in the figure legend, table legend, main text, or Methods section.

n/a	Confirmed	
☐	☒	The exact sample size (*n*) for each experimental group/condition, given as a discrete number and unit of measurement
☐	☒	A statement on whether measurements were taken from distinct samples or whether the same sample was measured repeatedly
☐	☒	The statistical test(s) used AND whether they are one- or two-sided<br>*Only common tests should be described solely by name; describe more complex techniques in the Methods section.*
☐	☒	A description of all covariates tested
☐	☒	A description of any assumptions or corrections, such as tests of normality and adjustment for multiple comparisons
☐	☒	A full description of the statistical parameters including central tendency (e.g. means) or other basic estimates (e.g. regression coefficient) AND variation (e.g. standard deviation) or associated estimates of uncertainty (e.g. confidence intervals)
☐	☒	For null hypothesis testing, the test statistic (e.g. *F*, *t*, *r*) with confidence intervals, effect sizes, degrees of freedom and *P* value noted<br>*Give P values as exact values whenever suitable.*
☒	☐	For Bayesian analysis, information on the choice of priors and Markov chain Monte Carlo settings
☒	☐	For hierarchical and complex designs, identification of the appropriate level for tests and full reporting of outcomes
☒	☐	Estimates of effect sizes (e.g. Cohen's *d*, Pearson's *r*), indicating how they were calculated

Our web collection on statistics for biologists contains articles on many of the points above.

Software and code

Policy information about availability of computer code

Data collection	Median fluorescence intensities were collected using xPONENTv4.3 from Luminex Corp. running on a windows XP. Glycan structures were drawn using ChemDraw version 19.1.0.8.
Data analysis	The data was analyzed using RStudio (v2022.07.1 Build 554.pro3) running R version 4. 1.2 and SAS v 9.4. Custom code was generated to analyze the data and is available here as a R Package on Github https://github.com/pmtran5884/Glycancc v 1.0 and on zenodo (doi: 10.5281/zenodo.7143430). Users can install the package along with required dependencies to run the analysis. the Github page contains instructions on installation of package and how to run the analysis. The instructions to install the package directly from github are provided on the page,t he reviewers are encourage to visit the page for installation. The attached zip file can be installed as a local file in R. List of all R packages used in this session are provided in supplementary tables and figures file. PCAtools and UMAP were utilized for PCA analysis.

For manuscripts utilizing custom algorithms or software that are central to the research but not yet described in published literature, software must be made available to editors and reviewers. We strongly encourage code deposition in a community repository (e.g. GitHub). See the Nature Portfolio guidelines for submitting code & software for further information.

Data

Policy information about availability of data

All manuscripts must include a data availability statement. This statement should provide the following information, where applicable:

- Accession codes, unique identifiers, or web links for publicly available datasets
- A description of any restrictions on data availability
- For clinical datasets or third party data, please ensure that the statement adheres to our policy

Samples and relevant clinical information were obtained from PAGODA and DAISY databases. The processed clinical and ACA data generated in this study have been

deposited on Zenodo under accession https://doi.org/10.5281/zenodo.7143430.

Field-specific reporting

Please select the one below that is the best fit for your research. If you are not sure, read the appropriate sections before making your selection.

- [x] Life sciences
- [ ] Behavioural & social sciences
- [ ] Ecological, evolutionary & environmental sciences

For a reference copy of the document with all sections, see nature.com/documents/nr-reporting-summary-flat.pdf

Life sciences study design

All studies must disclose on these points even when the disclosure is negative.

Sample size	No sample size calculation was performed. The full cohorts from the DAISY and PAGODA studies were used for discovery analysis which have historically provided strong effect sizes for analyses.
Data exclusions	No data exclusions.
Replication	We evaluated singleplex vs multiplex analysis. Day to day reproducibility analysis was also performed to ensure repeatability.
Randomization	All HLA based high risk individuals were selected for this analysis, matched for Age, Sex and HLA risk. In PAGODA study there was significant difference between the age in controls, non-progressors and progressors. DAISY data did not showed these differences due to matching for these co-variates.
Blinding	Investigators were blinded to study group during data collection and quality control.

Reporting for specific materials, systems and methods

We require information from authors about some types of materials, experimental systems and methods used in many studies. Here, indicate whether each material, system or method listed is relevant to your study. If you are not sure if a list item applies to your research, read the appropriate section before selecting a response.

Materials & experimental systems

n/a	Involved in the study	
[ ]	[x]	Antibodies
[x]	[ ]	Eukaryotic cell lines
[x]	[ ]	Palaeontology and archaeology
[x]	[ ]	Animals and other organisms
[ ]	[x]	Human research participants
[x]	[ ]	Clinical data
[x]	[ ]	Dual use research of concern

Methods

n/a	Involved in the study	
[x]	[ ]	ChIP-seq
[x]	[ ]	Flow cytometry
[x]	[ ]	MRI-based neuroimaging

Antibodies

Antibodies used	Secondary antibody (3ug/ml) used is PE (cat#2010-09) and biotinylated (Cat#2040-08) anti-human IgG raised in goat purchased from southern biotech, Alabama, USA. from PE-Conjugated Streptavidin (3ug/ml, Cat#LT-SAPE) was purchsed from One Lambda Inc, CA, USA.
Validation	no primary antibodies were used.

Human research participants

Policy information about studies involving human research participants

Population characteristics	The samples used in the study were obtained from participants of the Phenome and Genome of Diabetes Autoimmunity (PAGODA) and Diabetes Autoimmunity Study in the Young (DAISY) study. PAGODA study is a cross-sectional design and contained controls (n=278, 54% females, 18[7-41] years old), non-progressors (n=26, 50% females, 8[5-17] years old) and TlD patients (n=298, 55% females, 21[14-42] years old). 112 children at high risk for TlD from DAISY were followed for development of autoantibody or progression to TlD and they were divided into three comparison groups: progressors (n=47, 51% females, 6.6[4.1-8.5] years old), non-progressors (n=35, 49% females, 8.9[4.1-10.6] years old), and controls (n=30, 57% females, 8[5.4-11.1] years old). Progressors were children who progressed to type 1 diabetes (TlD). Non-progressors are children having at least two consecutive positive visits for islet autoantibodies by radiobinding (RBA) assay, but did not develop TlD at their last followup. participants from both the study were matched for HLA risk, gender, age and first degree relative status. Informed consent was obtained from all participants or their parents. Participatcipation in both PAGODA and DAISY study was voluntary. No compensation was provided to the participants. Recruitment for both studies identified genetically at risk newborns and followed them up to 20 years. Patients who developed T1D were cases and those who did not were controls. Hence, both studies are selected for individuals at high genetic risk of developing T1D which may play a role in decreasing the diversity of ACA with high genetic heritability (e.g. blood group antigens). We thus have lower statistical power for detecting differences in ACA levels for ACAs targeting glycans under human genetic control. Participants were recruited from Georgia, Florida, and Colorado. Hence, findings especially in relation to environmental exposures may not be generalizable to other populations.
Recruitment	Initial recruitment was from general public, without any selection bias whether self or otherwise. All samples analyzed in the study were from individuals with high risk HLA genes. the samples were matched for the HLA risk, age and gender to rule out any selection bias.
Ethics oversight	Institutional Review Boards at Augusta University and University of Colorado.

Note that full information on the approval of the study protocol must also be provided in the manuscript.
